# A Model of Basement Membrane-Associated Gene Signature Predicts Liver Hepatocellular Carcinoma Response to Immune Checkpoint Inhibitors

**DOI:** 10.1155/2023/7992140

**Published:** 2023-04-28

**Authors:** Jiajia Shen, Zhihong Wei, Lizhi Lv, Jingxiong He, Suming Du, Fang Wang, Ye Wang, Lin Ni, Xiaojin Zhang, Fan Pan

**Affiliations:** ^1^Department of Hepatobiliary Surgery, 900th Hospital of Joint Logistics Support Force (Fuzong Clinical Medical College) (Former Fuzhou General Hospital), Fuzhou, Fujian, China; ^2^Department of General Surgery, 900th Hospital of Joint Logistics Support Force (Fuzong Clinical Medical College) (Former Fuzhou General Hospital), Fuzhou, Fujian, China

## Abstract

Liver hepatocellular carcinoma (LIHC) is a highly lethal malignant tumor originating from the digestive system, which is a serious threat to human health. In recent years, immunotherapy has shown significant therapeutic effects in the treatment of LIHC, but only for a minority of patients. The basement membrane (BM) plays an important role in the occurrence and development of tumors, including LIHC. Therefore, this study is aimed at establishing a risk score model based on basement membrane-related genes (BMRGs) to predict patient prognosis and response to immunotherapy. First, we defined three patterns of BMRG modification (C1, C2, and C3) by consensus clustering of BMRG sets and LIHC transcriptome data obtained from public databases. Survival analysis showed that patients in the C2 group had a better prognosis, and Gene Set Variation Analysis (GSVA) revealed that the statistically significant pathways were mainly enriched in the C2 group. Moreover, we performed Weighted Correlation Network Analysis (WGCNA) on the above three subgroups and obtained 179 intersecting genes. We further applied function enrichment analyses, and the results demonstrated that they were mainly enriched in metabolism-related pathways. Furthermore, we conducted the LASSO regression analysis and obtained 4 BMRGs (MPV17, GNB1, DHX34, and MAFG) that were significantly related to the prognosis of LIHC patients. We further constructed a prognostic risk score model based on the above genes, which was verified to have good predictive performance for LIHC prognosis. In addition, we analyzed the correlation between the risk score and the tumor immune microenvironment (TIM), and the results showed that the high-risk scoring group tended to be in an immunosuppressed status. Finally, we investigated the relationship between the risk score and LIHC immune function. The results demonstrated that the risk score was closely related to the expression levels of multiple immune checkpoints. Patients in the low-risk group had significantly higher IPS scores, and patients in the high-risk group had lower immune escape and TIDE score. In conclusion, we established a novel risk model based on BMRGs, which may serve as a biomarker for prognosis and immunotherapy in LIHC.

## 1. Introduction

Liver cancer is a highly lethal malignant tumor that seriously threatens human physical and mental health. Liver hepatocellular carcinoma (LIHC) is the most common pathological type of liver cancer, accounting for more than 90% of all cases [1]. Currently, the most common treatment options for LIHC are surgery, ablation, microwave ablation, cryotherapy ablation, percutaneous ethanol injection, and noncatheter-based therapies, but the 5-year survival rate is less than 20% [2–4]. It is now widely recognized that the poor prognosis of LIHC is due to the lack of appropriate prognostic biomarkers [5]. Therefore, it is crucial to develop a model to identify high-risk patients and enable personalized medicine for LIHC patients.

In recent years, with the deepening understanding of the pathogenesis of the tumor, a variety of immune checkpoint inhibitors (ICIs) based on immune checkpoints have gradually become the focus of LIHC treatment. At present, the PD1/PD-L1 antibody is widely used in the immunotherapy of LIHC and has achieved significant treatment effects [6]. It has been confirmed that the tumor immune environment (TIM) is key to the immunotherapeutic effect of ICIs [7]. Therefore, it is particularly critical to clarify the specific regulation mechanism of regulating the TIM of LIHC.

The structure of the basement membrane (BM) plays a key role in the occurrence and development of malignant tumors [8–10]. Under normal physiological conditions, the BM is a sheet-like structure under the epithelial cells, of which laminin and type IV collagen are its main structural components [11]. The BM not only resists mechanical stress and maintains tissue shape but also regulates the adhesion and migration of various cells, including immune cells [12]. However, under tumor conditions, the structure of the BM is destroyed, resulting in the loss of its original shape and function, which in turn causes abnormal migration of tumor cells and various immune cells [8, 10, 13]. Epithelial-mesenchymal transition (EMT) of the basement membrane promotes the transfer of tumor cells through the lymphatic vasculature in an intravasation and extravasation manner [14]. The products of tumor metabolism can induce changes in the structural components of the BM, thereby enhancing the metastatic ability of tumors [15]. Other studies have confirmed that the migration ability of T cells in the dense collagen matrix area around the tumor nest is significantly reduced, and the reduction of the collagen matrix density in the BM will enhance the infiltration of T cells in the tumor [16]. Although this change has little effect on tumor growth, it does improve response to anti-PD1 therapy [17, 18]. In 2022, Jayadev et al. applied bioinformatics and in vivo experiments to define more than 200 genes related to BM, such as LAMA5, MPZL2, and MATN2 [19]. Therefore, a better understanding of the role of the basement membrane may lead to new and promising treatments for LIHC.

In this study, we first obtained the transcriptome data of LIHC from the TCGA database and then further analyzed and screened 4 basement membrane-related genes (BMRGs) that were significantly associated with the prognosis of LIHC. Furthermore, we constructed a prognostic risk model by screening the BMRGs and confirmed that the model has good predictive capacity for the prognosis of LIHC patients. Finally, we further evaluated the differences in the risk score of this model for immune cell infiltration and immunotherapy response. Our study provides a novel research direction for the monitoring of prognosisand evaluation of immunotherapy in LIHC.

## 2. Materials and Methods

### 2.1. Identification of BMRGs and Collection of LIHC Transcriptome Data

First, we obtained 222 basement membrane-related gene sets from previous studies. Next, we used the public database TCGA to download the LIHC transcriptome information. The survival information of the LIHC samples was merged with the transcriptome data, and finally, 342 LIHC samples with survival information were obtained.

### 2.2. Construction of Risk Scoring Model

We obtained the basement membrane-related gene sets associated with patient prognosis by the LASSO Cox regression analysis. The risk score for each LIHC patient was calculated according to an established formula. Risk score = (*β*_*i*_^∗^ Exp_*i*_), where Exp_*i*_ represents the expression level of each gene and *β*_*i*_ represents the coefficient of each gene [20]. ROC curves were used to evaluate the accuracy of the predictive power of each dataset.

### 2.3. Consensus Clustering of 222 Basement Membrane-Related Genes by NMF Algorithm

We applied the NMF algorithm for consensus clustering to identify different classification patterns based on the expression of 222 BMRGs. Then, the optimal number of clusters is selected according to the cooccurrence coefficient, dispersion coefficient, and silhouette coefficient [21].

### 2.4. Analysis of Immune Cell Infiltration in LUAD

We applied CIBERSORT to assess the correlation between the high- and low-risk scores and the proportion of immune cell infiltration. CIBERSORT relies on a gene expression matrix file (named LM22), which can specifically identify specific genes in immune cells. The expression of this specific gene can analyze immune cells in tissues and identify human hematopoietic cell phenotypes [22].

### 2.5. IPS, ESTIMATE, and TIDE

The immunophenoscore (IPS) is a predictor of response to anti-CTLA-4 and anti-PD1 therapy by quantifying tumor immunogenicity, immunomodulators, effector cells, and suppressor cells. This method obtains the final IPS score by the weighted quantification of the above components [23]. ESTIMATE (estimation of stromal and immune cells in malignant tumor organizations using expression data) is a novel algorithmic algorithm that infers tumor cell structure and distinct infiltrating normal cells from uniquely characterized genes in the transcriptional profile of cancer tissues [24]. In this study, by using the ESTIMATE algorithm, we calculated the immune and stromal scores to predict the correlation of risk scores with immune and stromal levels. The tumor immune dysfunction and exclusion (TIDE) is an algorithm used to predict response to immune checkpoint inhibitors. Low TIDE scores represent weaker immune evasion, and these patients may show a stronger response to immunotherapy, while high TIDE scores represent strong immune evasion, and these patients are less responsive to immunotherapy [25].

### 2.6. Statistics

In this study, gene expression data from TCGA database were analyzed using Student's *t*-test. Correlation analysis of Spearman and Pearson was used to assess in the TISdb database. The expression of ADAR1 was correlated with the abundance scores of immune cells assessed using Spearman's correlation analysis. All analyses were performed with the R software (version 4.1.1, http://www.r-project.org) loaded with the R packages (“ggplot2,” “ggpubr,” “limma,” “survival,” “survminer,” “clusterProfiler,” “ESTIMATE,” “enrichplot,” and “forestplot”), and the results were visualised. *p* value < 0.05 was considered statistically significant.

## 3. Results

### 3.1. Consensus Clustering Analysis of BMRGs in LIHC by NMF Algorithm

**Figure 1:**
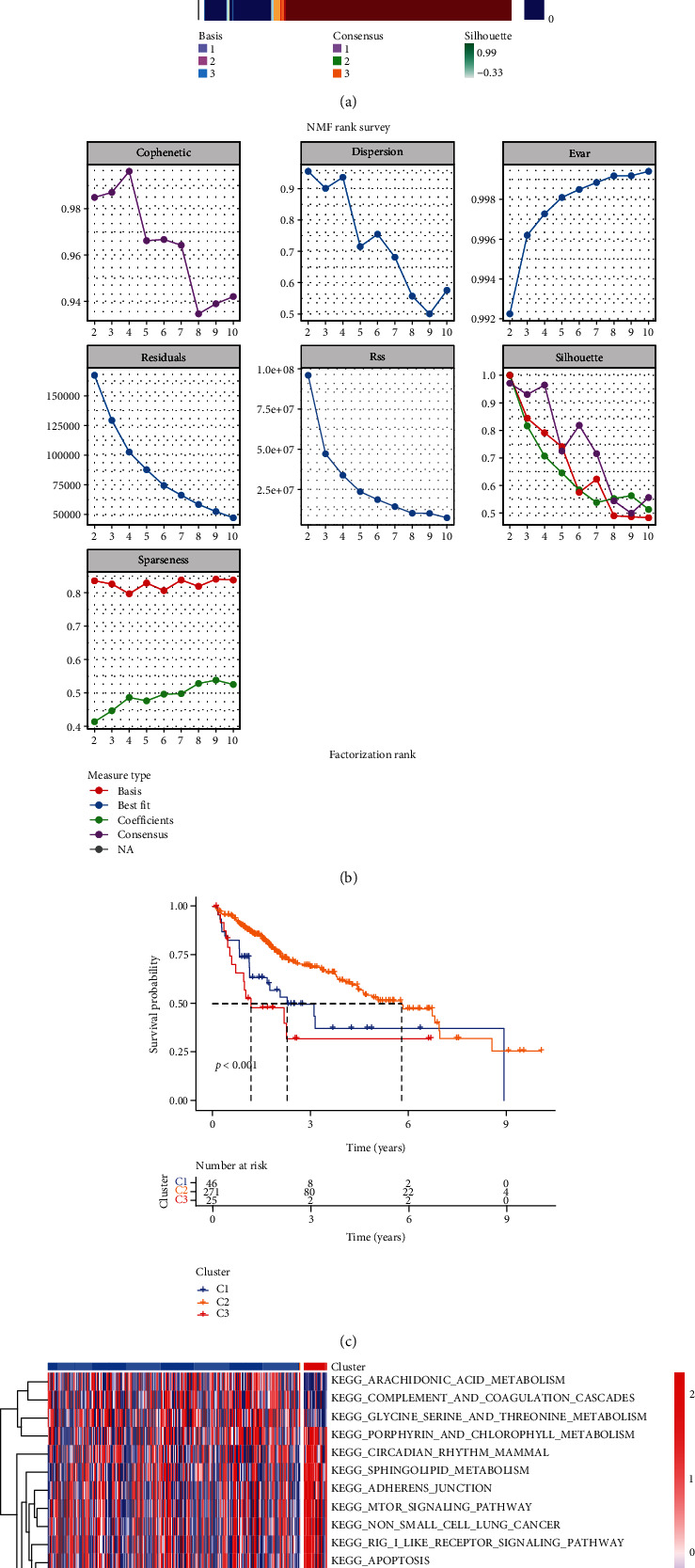
BMRG consensus cluster and relevant biological pathway. (a) Nonnegative matrix factorization (NMF) clustering was conducted, and three subgroups were identified as the optimal value for consensus clustering. (b) Factorization rank for *k* = 2‐10. (c) The Kaplan-Meier curves of overall survival (OS) for 342 LIHC patients in TCGA cohort with different BMRG clusters. The numbers of C1, C2, and C3 patients are 46, 271, and 25, respectively (log-rank test). (d) GSVA analysis heatmap for different clusters.

The structure of the BM regulates the migration of tumor and immune cells in a variety of malignancies [26, 27]. First, we applied the consensus clustering analysis of the NMF algorithm to stratify 222 basement membrane-related genes into 9 subtypes (Supplementary Figure 1). As seen in the cophenetic, the curve decline was most pronounced when all samples were separated into type 3, so we identified three distinct clusters of modification modes. The three different patterns of cluster distribution were cluster 1 (146 cases, named C1), cluster 2 (271 cases, named C2), and cluster 3 (25 cases, named C3) (Figures 1(a) and 1(b)). Next, we performed survival analysis, which showed that C2 had a better survival prognosis, whereas C3 had the worst prognosis (Figure 1(c)). In addition, we further conducted the GSVA on C2 and C3, and the results showed that a variety of pathways were abnormally enriched in the samples of C3 (Figure 1(d)).

### 3.2. WGCNA and Difference Analysis Based on Different Typing of BMRGs in LIHC

**Figure 2:**
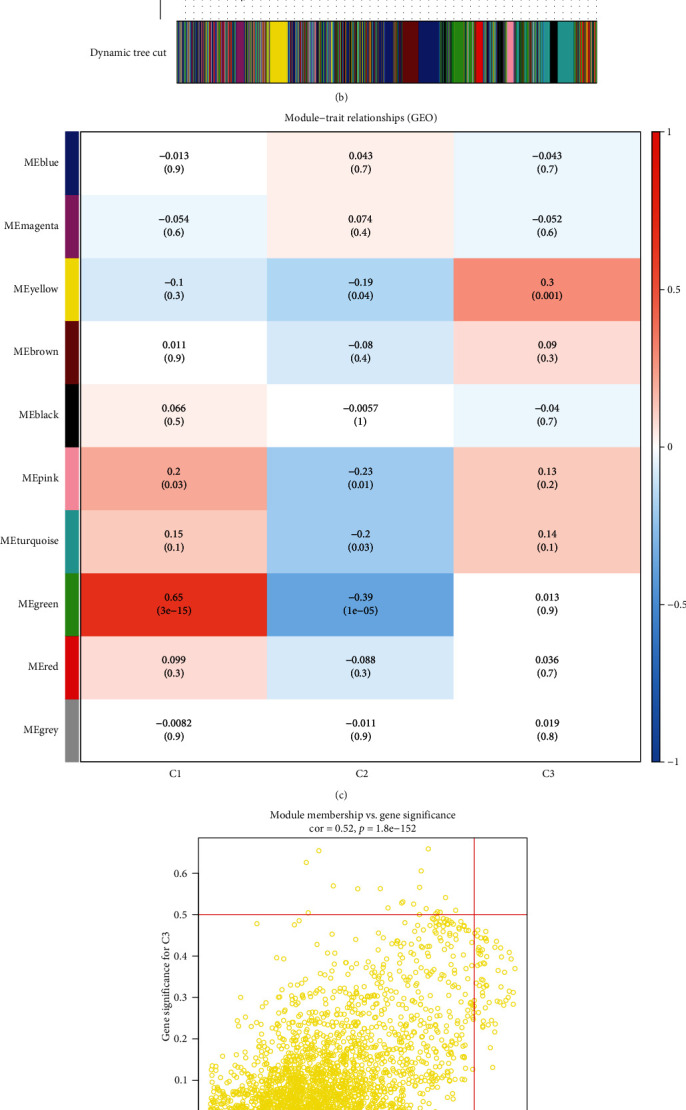
The WGCNA of the NMF phenotypes of BM. (a, b) Detailed results of the weighted gene coexpression network analysis. (c) The relationship of module features with the consensus subgroups was assessed by ten gene modules obtained from WGCNA. (d) The results of module-feature relationship analysis between the yellow module and the consensus subgroup C3. (e) The results of module-feature relationship analysis between the green module and the consensus subgroup C1.

**Figure 3:**
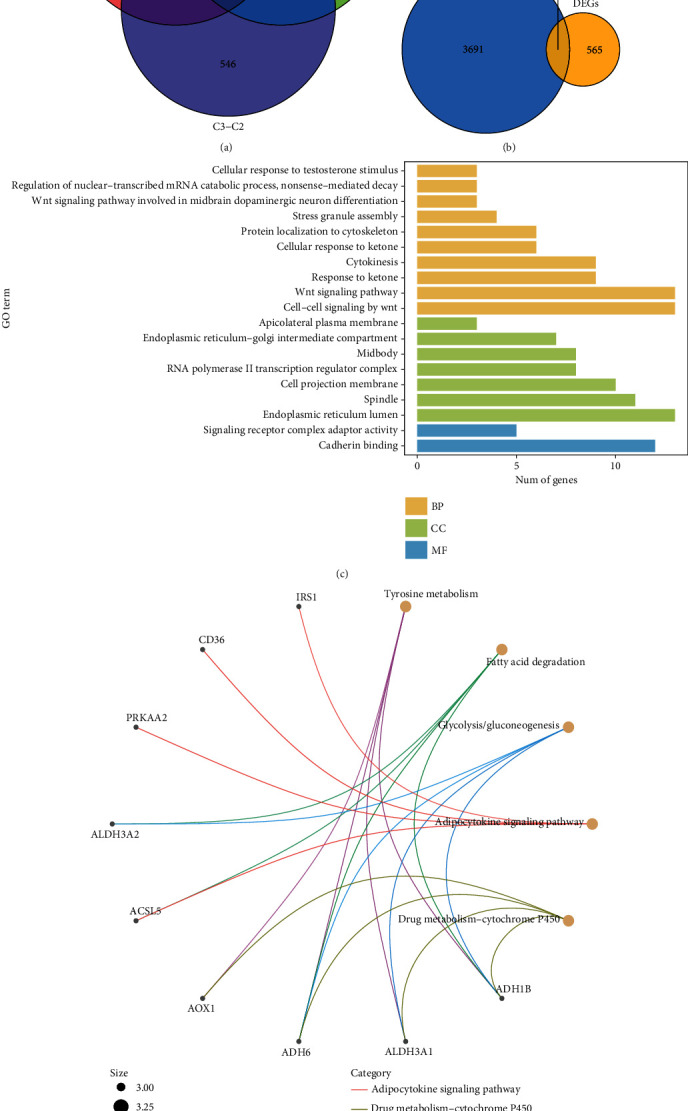
Identification and functional analysis of BMRGs. (a) Differential genes for the three molecular clusters of NMF. (b) Venn diagram of WGCNA module genes with differential genes. (c) GO functional analysis of intersecting genes. (d) KEGG functional analysis of intersecting genes.

Given the obtained 3 different subtypes of LIHC based on BMRGs, we applied the weighted gene coexpression network analysis (WGCNA) to analyze the above subtypes. In this study, we chose 6 as the optimal threshold (Figure 2(a)). Based on the WGCNA results, we obtained 10 coexpressed gene modules (Figure 2(b)). The yellow module was significantly correlated with the worst prognosis C3, and the green module was closely correlated with the worst prognosis C1 (Figure 2(c)). As shown in Figures 2(d) and 2(e), there was a significant correlation between the gene sets within these two modules and the signatures in each type. In addition, we performed the differentially expressed genes (DEGs) analysis on each of the three subgroups C1, C2, and C3 and obtained a total of 745 genes with statistical significance (Figure 3(a)). Furthermore, based on the 3770 coexpressed genes obtained by the green and yellow modules, we took the intersection with the abovementioned differential genes and finally obtained a total of 179 intersecting genes (Figure 3(b)). Finally, we applied GO and KEGG enrichment analyses on these 179 genes, and the results showed that they were mainly enriched in related metabolic pathways, such as tyrosine metabolism, fatty acid metabolism glycolysis, and adipokine signaling pathway (Figures 3(c) and 3(d)).

### 3.3. Construction and Validation of BMRG Risk Scoring Model

**Figure 4:**
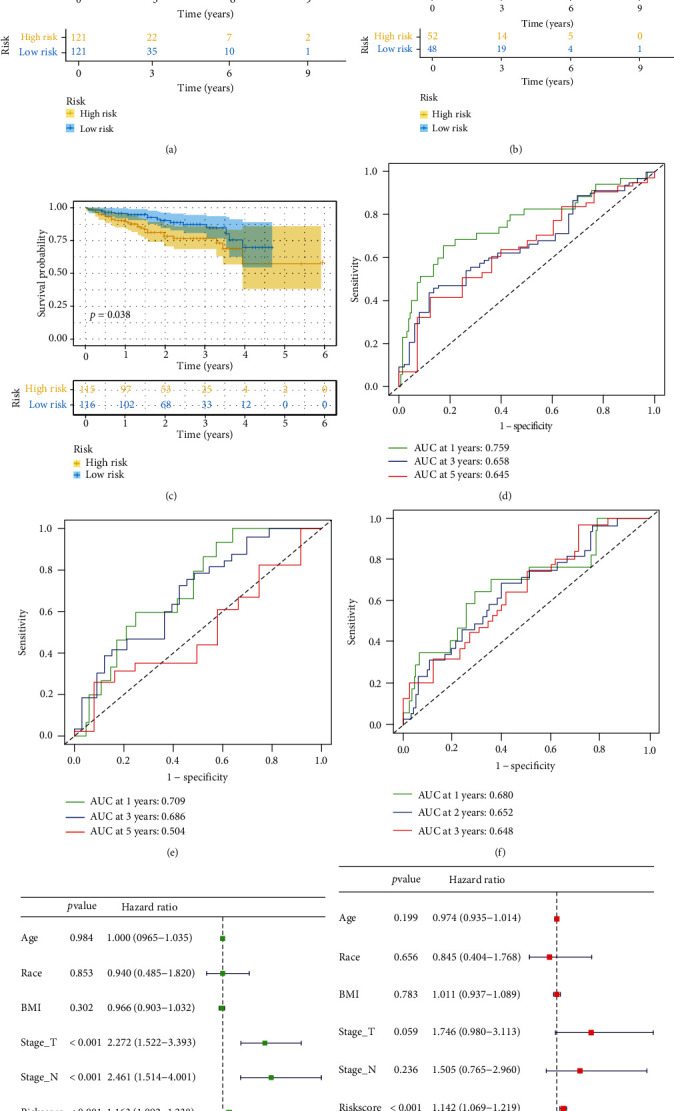
Construction and verification of the BMRG risk model by LASSO Cox regression analysis. (a–c) The Kaplan-Meier curves for patients with the high- and low-BMRG subgroups: (a) train cohort, (b) test cohort, and (c) ICGC cohort. (d–f) ROC curves showing the predictive efficiency of the BMRG risk scores for 1-year, 3-year, and 5-year survival: (d) train set, (e) test set, and (f) ICGC validation set. (g) Univariate analysis of risk scores of BMRGs and clinicopathological characteristics of LIHC. (h) Multivariate analysis of risk scores of BMRGs and clinicopathological characteristics of LIHC.

We first performed the LASSO regression analysis on the obtained 179 genes and screened 4 basement membrane-related genes (Supplementary Figure 2). Next, we randomly divided the LIHC samples in TCGA into two cohorts, namely, the training cohort and the validation cohort, at a ratio of 7 : 3, while using the ICGC-LIHC cohort as the external validation cohort. Furthermore, we constructed a LIHC risk prognostic model with basement membrane characteristics using the four genes obtained above. In the training, validation, and external validation cohorts, high-risk patients had significantly worse outcomes than low-risk patients (Figures 4(a)–4(c) and Supplementary Figure 3). In addition, we used ROC curves to evaluate the predictive power of the BMRG risk model, and the results showed that the AUCs of each cohort at 1, 3, and 5 years were 0.759, 0.658, and 0.645 (training cohort); 0.709, 0.686, and 0.504 (validation cohort); and 0.680, 0.680, 0.652, and 0.648 (external validation cohort) (Figures 4(d)–4(f)), and these results show that the model has good predictive performance. Finally, we applied the univariate and multivariate Cox regression analyses on the risk score combined with each clinical feature, and the results revealed that the risk prognostic model based on BMRG could be used as an independent prognostic factor (Figures 4(g) and 4(h)).

### 3.4. Correlation Analysis between BMRG Risk Score and LIHC Immune Microenvironment

**Figure 5:**
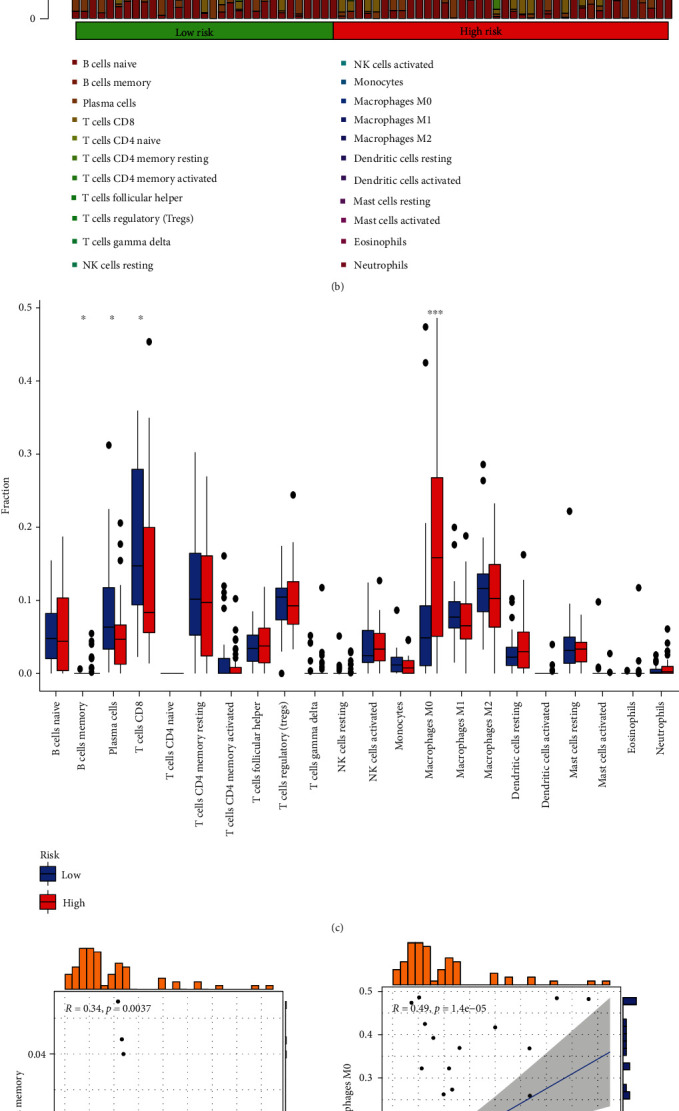
The relationship between the BMRG risk score and the TIM in LIHC. (a) Correlation between the BMRG risk score and the TME score. (b) The proportion of tumor-infiltrating immune cells in the BMRG high- and low-risk cohort via the CIBERSORT algorithm. (c) Comparison of different immune cell infiltrations under high and low risk scores. (d–g) Linear relationship between BMRG risk score and immune cells.

The TIM is closely related to tumor immune escape. To clarify their complex relationship, we evaluated the TIM of LIHC by the ESTIMATE algorithm and observed that the low-risk cohort had significantly higher stromal scores than the high-risk cohort (Figure 5(a)). Next, we assessed the correlation between the infiltration abundance of immune cells and the risk score by the CIBERSORT algorithm and presented them in the form of heatmaps and boxplots. The results showed that the infiltrating abundance of CD8^+^ T cells and plasma cells was higher in the low-risk cohort than in the high-risk group (Figures 5(b) and 5(c)). As shown in Figures 5(d)–5(g), we further analyzed the correlation between the risk score and the degree of immune cell infiltration, and the results showed that memory B cells, M0 macrophages, and dendritic cells were positively associated with the risk score, while CD8^+^ was negatively associated with the risk score. The above results strongly suggested that the risk score of this model is closely related to the TIM of LIHC patients.

### 3.5. The Role of the BMRG Risk Score in Predicting Response to Immunotherapy

**Figure 6:**
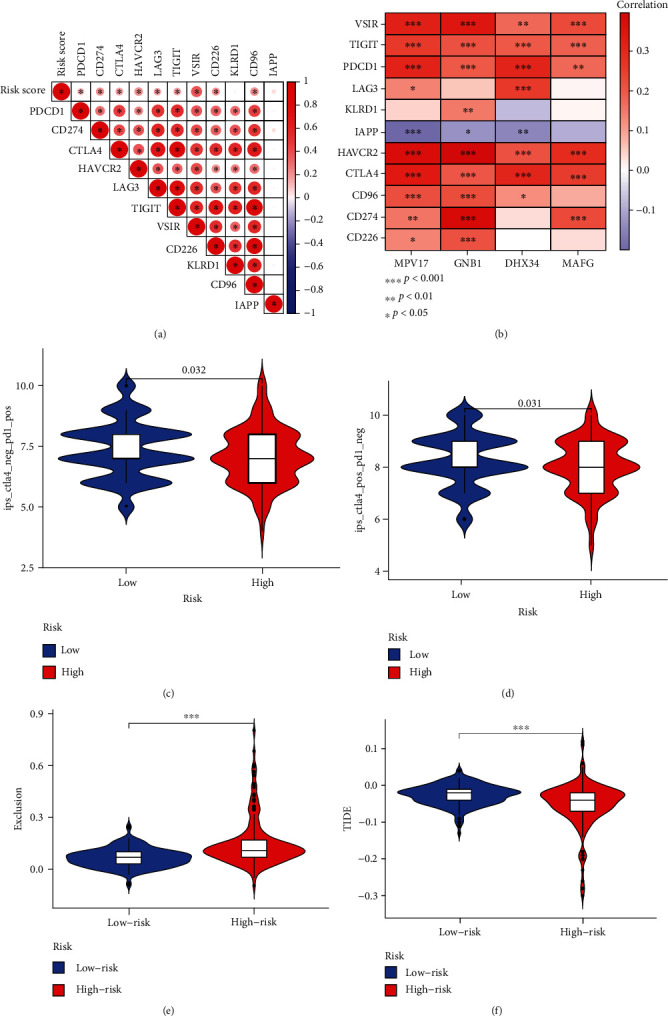
The BMRG risk score predicts immunotherapeutic benefits. (a) Association of BMRG risk scores with immune checkpoints. (b) Association of four BMRGs with eleven immune checkpoints. (c, d) The proportion of patients with clinical response to anti-PD1 and anti-CTLA4 immunotherapy in the low- or high-BMRG-risk group. (e) Differences in immune escape score between the high- and low-risk groups. (f) Differences in TIDE scores between the high- and low-risk groups.

Immune checkpoints are important receptors that regulate immune cell function and are important predictors for evaluating immunotherapy response [28] Therefore, we evaluated the association of 11 immune checkpoints with risk scores of BMRGs, and the results showed that risk scores were positively correlated with multiple immune checkpoints (Figure 6(a)). Next, we analyzed the relationship between the 4 BMRGs in the model and immune checkpoints, and the results demonstrated that IAPP was negatively correlated with these genes, while other immune checkpoints were positively correlated with 4 genes (Figure 6(b)). Given the strong correlation between BMRG scores and immune checkpoints, we further investigated whether the risk scores of BMRGs could predict the response of LIHC patients to ICIs. The IPS scoring system is widely applied to assess response to immunotherapy at present. In this study, we found that the IPS scores of PD1-positive and CTLA4-positive patients were significantly elevated in the low-risk group, and the IPS scores of PD1-negative and CTLA4-positive patients were also significantly elevated in the low-risk group (Figures 6(c) and 6(d)). Finally, we demonstrated that high-risk patients had stronger immune evasion and worse TIDE scores (Figures 6(e) and 6(f)). These findings indirectly indicated that risk scoring models based on BMRGs can be used to assess response to immunotherapy.

### 3.6. GSEA of BMRG Risk Model

**Figure 7:**
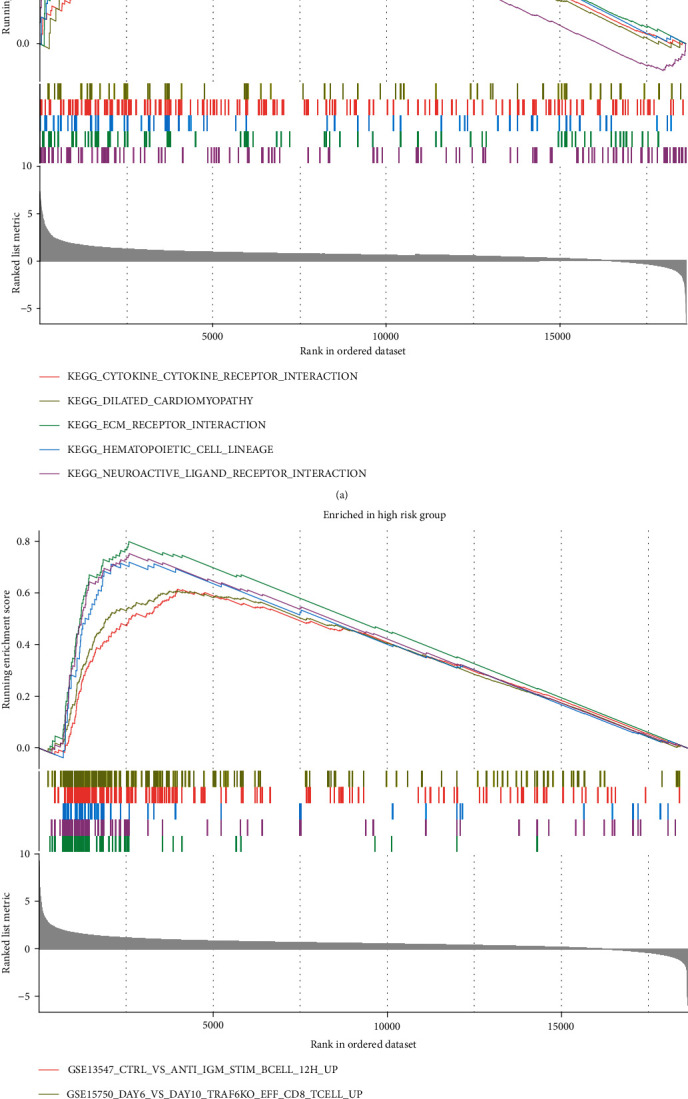
GSEA of BMRGs. (a) GSEA of KEGG gene set for the high- and low-risk cohorts of BMRGs. (b) GSEA of immune gene set for the high- and low-risk cohorts of BMRGs.

Our previous data suggested that the BMRG risk score is closely related to the TIM of LIHC. To further elucidate the underlying mechanism, we performed GSEA by differentially expressed genes between the high- and low-risk cohorts. The results of the KEGG enrichment analysis showed that the high-risk cohorts were mainly enriched in cytokine receptor interaction, extracellular matrix receptor interaction, and neuroligand-receptor interaction pathways (Figure 7(a)). Meanwhile, the enrichment results of the immune gene set showed that the high-risk cohort was mainly enriched in B cells, CD8^+^ T cells, NK cells, and monocytes (Figure 7(b)).

## 4. Discussion

Recurrence and metastasis are the main causes of treatment failure in LICH. Different from traditional treatments, immunotherapy is a promising treatment for LIHC. BM structure plays an important role in immune cell migration and is closely related to prognosis [29, 30]. In this study, we first performed consensus clustering of BMRGs using the NMF algorithm to classify all samples into three patterns. In addition, through WGCNA and differential gene analysis, the intersection between the two was further taken to obtain a differential gene set. Moreover, LASSO regression analysis was performed on the obtained differential gene set, and a prognostic risk score model based on BMRGs was constructed. Its predictive ability was further verified. Finally, we found that a risk score model based on BMRGs could have good predictive power for the immune microenvironment and immunotherapy.

In recent years, a variety of prognostic risk models based on cell-related functional genes have been developed, which provide favorable help for the prognosis assessment of various malignant tumors. Luo et al. analyzed the expression of ferroptosis-related genes in LIHC from public databases and constructed a corresponding prognostic model. The AUC areas for the model at 1, 3, and 5 years were 0.6838, 0.694, and 0.559, respectively [31]. Yu et al. constructed a prognostic model with good predictive ability by extracting the pyroptotic genes in LIHC. The AUC areas for 1, 3, and 5 years were 0.748, 0.732, and 0.603, respectively [32]. In this study, the AUC of our prognostic model was 0.759, 0.658, and 0.654 at 1, 3, and 5 years, respectively. Compared with previous related functional gene set models, the model established in this study has higher predictive performance.

The BM plays an important role in both physiological and pathological states, so the set of genes involved in regulating the structure of the basement membrane is particularly important. In this study, we found that the risk model based on BMRGs was closely related to the immune cell infiltration of LIHC. Meanwhile, we also found that the high-risk score of this model suggested low responsiveness to tumor immunotherapy. These evidences strongly indicated that the BMRGs not only regulate the infiltration of leukocytes but may also be related to the checkpoint function of multiple immune cells. For these surprising findings, we intend to further develop in vitro and in vivo use in follow-up studies to support the above inferences.

In this study, we revealed the important role of BMRGs in LIHC, which also provides new directions for the treatment of LIHC, but there are still many shortcomings. First, all LIHC data in this study was derived from public databases and lacked validation in vivo and in vitro. In addition, the biological molecular mechanism of various genes in BMRGS has not been explored, which greatly limits its accuracy.

In conclusion, our study revealed that BM is closely related to LIHC progression. We provided a novel BMRG risk model to predict LIHC patients' survival. In addition, our established model can provide guidance on the immune microenvironmental status of LIHC and the efficacy of immunotherapy. We firmly believe that the model based on BMRGs has excellent application prospects after further verification.

## Data Availability

All data and result in this study are available from the corresponding author for reasonable request.

## Supplementary Materials

Supplementary Materials Supplementary Figure 1: nonnegative matrix decomposition (NMF) clustering was performed, showing a total of eight subgroups to determine the best values for consensus clustering. Supplementary Figure 2: construction of the BM-related risk model by the LASSO Cox regression analysis. (A) The partial likelihood deviations of the variables revealed by the LASSO regression model. The red dots indicate the partial likelihood of the deviation values, the gray line indicates the standard error (SE), and the two vertical dashed lines on the left and right represent the minimum standard and the optimal value of the 1-SE standard, respectively. (B) Coefficient profiles of the 179 prognosis-related BM-related genes via LASSO Cox regression analysis. Supplementary Figure 3: distribution of risk curves and number of patients in BMRG risk score. (A, B) Train set and (C, D) test set.

## References

[1] Llovet J. M., Kelley R. K., Villanueva A. (2021). Hepatocellular carcinoma. *Nature Reviews Disease Primers*.

[2] Sugawara Y., Hibi T. (2021). Surgical treatment of hepatocellular carcinoma. *Bioscience Trends*.

[3] Arciero C. A., Sigurdson E. R. (2006). Liver-directed therapies for hepatocellular carcinoma. *Journal of the National Comprehensive Cancer Network*.

[4] Chino F., Stephens S. J., Choi S. S. (2018). The role of external beam radiotherapy in the treatment of hepatocellular cancer. *Cancer*.

[5] Nault J. C., Villanueva A. (2021). Biomarkers for hepatobiliary cancers. *Hepatology*.

[6] Xu F., Jin T., Zhu Y., Dai C. (2018). Immune checkpoint therapy in liver cancer. *Journal of Experimental & Clinical Cancer Research*.

[7] Lei X., Lei Y., Li J. K. (2020). Immune cells within the tumor microenvironment: biological functions and roles in cancer immunotherapy. *Cancer Letters*.

[8] Liu X., Qiao Y., Chen J., Ge G. (2022). Basement membrane promotes tumor development by attenuating T cell activation. *Journal of Molecular Cell Biology*.

[9] Grant D. S., Kibbey M. C., Kinsella J. L., Cid M. C., Kleinman H. K. (1994). The role of basement membrane in angiogenesis and tumor growth. *Pathology, Research and Practice*.

[10] Terranova V. P., Hujanen E. S., Martin G. R. (1986). Basement membrane and the invasive activity of metastatic tumor cells. *Journal of the National Cancer Institute*.

[11] Randles M. J., Humphries M. J., Lennon R. (2017). Proteomic definitions of basement membrane composition in health and disease. *Matrix Biology*.

[12] Srivastava A., Pastor-Pareja J. C., Igaki T., Pagliarini R., Xu T. (2007). Basement membrane remodeling is essential for drosophila disc eversion and tumor invasion. *Proceedings of the National Academy of Sciences of the United States of America*.

[13] Hallmann R., Zhang X., Di Russo J. (2015). The regulation of immune cell trafficking by the extracellular matrix. *Current Opinion in Cell Biology*.

[14] Banerjee S., Lo W. C., Majumder P. (2022). Multiple roles for basement membrane proteins in cancer progression and EMT. *European Journal of Cell Biology*.

[15] Niu D., Luo T., Wang H., Xia Y., Xie Z. (2021). Lactic acid in tumor invasion. *Clinica Chimica Acta*.

[16] Kowalczyk D. W., Wlazlo A. P., Blaszczyk-Thurin M., Xiang Z. Q., Giles-Davis W., Ertl H. C. (2001). A method that allows easy characterization of tumor-infiltrating lymphocytes. *Journal of Immunological Methods*.

[17] Peng D. H., Rodriguez B. L., Diao L. (2020). Collagen promotes anti-PD-1/PD-L1 resistance in cancer through LAIR1-dependent CD8^+^ T cell exhaustion. *Nature Communications*.

[18] Mariathasan S., Turley S. J., Nickles D. (2018). TGF*β* attenuates tumour response to PD-L1 blockade by contributing to exclusion of T cells. *Nature*.

[19] Jayadev R., Morais M., Ellingford J. M. (2022). A basement membrane discovery pipeline uncovers network complexity, regulators, and human disease associations. *Science Advances*.

[20] Liang J. Y., Wang D. S., Lin H. C. (2020). A novel ferroptosis-related gene signature for overall survival prediction in patients with hepatocellular carcinoma. *International Journal of Biological Sciences*.

[21] Wang T., Dang N., Tang G. (2022). Integrating bulk and single-cell RNA sequencing reveals cellular heterogeneity and immune infiltration in hepatocellular carcinoma. *Molecular Oncology*.

[22] Bi K. W., Wei X. G., Qin X. X., Li B. (2020). BTK has potential to be a prognostic factor for lung adenocarcinoma and an indicator for tumor microenvironment remodeling: a study based on TCGA data mining. *Frontiers in Oncology*.

[23] Wu J., Li L., Zhang H. (2021). A risk model developed based on tumor microenvironment predicts overall survival and associates with tumor immunity of patients with lung adenocarcinoma. *Oncogene*.

[24] Ma Q., Chen Y., Xiao F. (2021). A signature of estimate-stromal-immune score-based genes associated with the prognosis of lung adenocarcinoma. *Translational Lung Cancer Research*.

[25] Wang Q., Li M., Yang M. (2020). Analysis of immune-related signatures of lung adenocarcinoma identified two distinct subtypes: implications for immune checkpoint blockade therapy. *Aging*.

[26] Sweeney T. M., Kibbey M. C., Zain M., Fridman R., Kleinman H. K. (1991). Basement membrane and the SIKVAV laminin-derived peptide promote tumor growth and metastases. *Cancer Metastasis Reviews*.

[27] Hagedorn E. J., Sherwood D. R. (2011). Cell invasion through basement membrane: the anchor cell breaches the barrier. *Current Opinion in Cell Biology*.

[28] Li B., Chan H. L., Chen P. (2019). Immune checkpoint inhibitors: basics and challenges. *Current Medicinal Chemistry*.

[29] Bremnes R. M., Donnem T., Al-Saad S. (2011). The role of tumor stroma in cancer progression and prognosis: emphasis on carcinoma-associated fibroblasts and non-small cell lung cancer. *Journal of Thoracic Oncology*.

[30] Grzelczyk W. L., Szemraj J., Jozefowicz-Korczynska M. (2016). The matrix metalloproteinase in larynx cancer. *Postȩpy Higieny i Medycyny Doświadczalnej*.

[31] Luo L., Yao X., Xiang J., Huang F., Luo H. (2022). Identification of ferroptosis-related genes for overall survival prediction in hepatocellular carcinoma. *Scientific Reports*.

[32] Yu H., Bai X., Zheng W. (2022). Identification of the pyroptosis-related prognosis gene signature and immune infiltration in hepatocellular carcinoma. *Disease Markers*.

