# Supplementary figures and images for: A Model of Basement Membrane-Associated Gene Signature Predicts Liver Hepatocellular Carcinoma Response to Immune Checkpoint Inhibitors
Source: Mediators Inflamm. 2023 Apr 28;2023:7992140. doi: 10.1155/2023/7992140 (PMC10162867; doi:10.1155/2023/7992140)

A

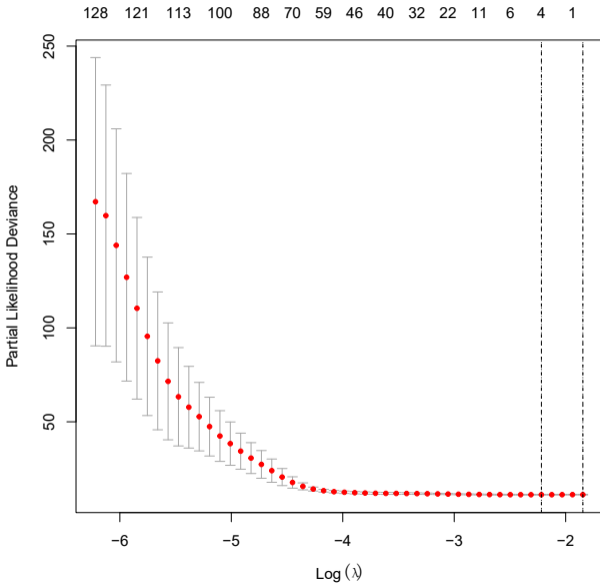

B

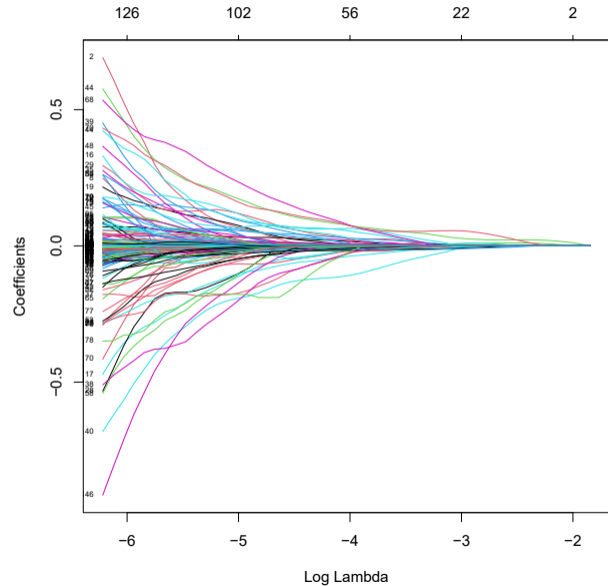

Supplement: Supplementary Materials — Supplementary Figure 1: nonnegative matrix decomposition (NMF) clustering was performed, showing a total of eight subgroups to determine the best values for consensus clustering. Supplementary Figure 2: construction of the BM-related risk model by the LASSO Cox regression analysis. (A) The partial likelihood deviations of the variables revealed by the LASSO regression model. The red dots indicate the partial likelihood of the deviation values, the gray line indicates the standard error (SE), and the two vertical dashed lines on the left and right represent the minimum standard and the optimal value of the 1-SE standard, respectively. (B) Coefficient profiles of the 179 prognosis-related BM-related genes via LASSO Cox regression analysis. Supplementary Figure 3: distribution of risk curves and number of patients in BMRG risk score. (A, B) Train set and (C, D) test set. [file 7992140.f1.zip › Supplementary Figure 2.pdf]

A

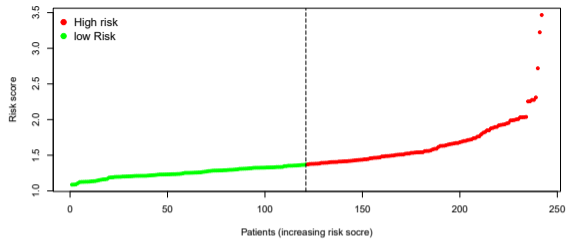

B

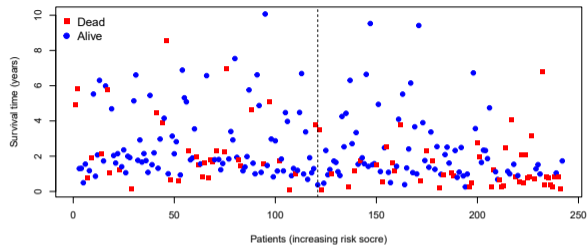

C

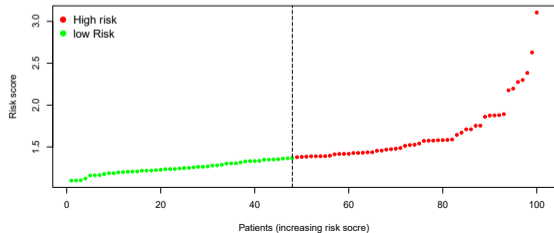

D

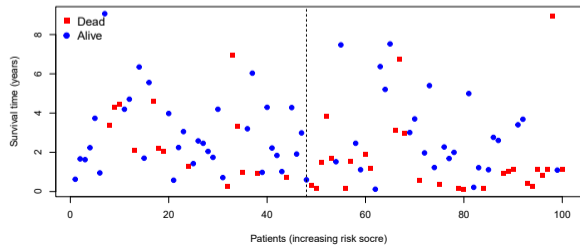

Supplement: Supplementary Materials — Supplementary Figure 1: nonnegative matrix decomposition (NMF) clustering was performed, showing a total of eight subgroups to determine the best values for consensus clustering. Supplementary Figure 2: construction of the BM-related risk model by the LASSO Cox regression analysis. (A) The partial likelihood deviations of the variables revealed by the LASSO regression model. The red dots indicate the partial likelihood of the deviation values, the gray line indicates the standard error (SE), and the two vertical dashed lines on the left and right represent the minimum standard and the optimal value of the 1-SE standard, respectively. (B) Coefficient profiles of the 179 prognosis-related BM-related genes via LASSO Cox regression analysis. Supplementary Figure 3: distribution of risk curves and number of patients in BMRG risk score. (A, B) Train set and (C, D) test set. [file 7992140.f1.zip › Supplementary Figure 3.pdf]
